# Huntingtin and the Synapse

**DOI:** 10.3389/fncel.2021.689332

**Published:** 2021-06-15

**Authors:** Jessica C. Barron, Emily P. Hurley, Matthew P. Parsons

**Affiliations:** Division of Biomedical Sciences, Faculty of Medicine, Memorial University, St. John’s, NL, Canada

**Keywords:** Huntington disease, Huntingtin, synaptic plastcity, endocytosis, exocytosis, intracellular tranport, autophagy, excitotoxicity

## Abstract

Huntington disease (HD) is a monogenic disease that results in a combination of motor, psychiatric and cognitive symptoms. HD is caused by a CAG trinucleotide repeat expansion in the huntingtin (*HTT*) gene, which results in the production of a pathogenic mutant HTT protein (mHTT). Although there is no cure at present for HD, a number of RNA-targeting therapies have recently entered clinical trials which aim to lower mHTT production through the use of antisense oligonucleotides (ASOs) and RNAi. However, many of these treatment strategies are non-selective in that they cannot differentiate between non-pathogenic wild type HTT (wtHTT) and the mHTT variant. As HD patients are already born with decreased levels of wtHTT, these genetic therapies may result in critically low levels of wtHTT. The consequence of wtHTT reduction in the adult brain is currently under debate, and here we argue that wtHTT loss is not well-tolerated at the synaptic level. Synaptic dysfunction is an extremely sensitive measure of subsequent cell death, and is known to precede neurodegeneration in numerous brain diseases including HD. The present review focuses on the prominent role of wtHTT at the synapse and considers the consequences of wtHTT loss on both pre- and postsynaptic function. We discuss how wtHTT is implicated in virtually all major facets of synaptic neurotransmission including anterograde and retrograde transport of proteins to/from terminal buttons and dendrites, neurotransmitter release, endocytic vesicle recycling, and postsynaptic receptor localization and recycling. We conclude that wtHTT presence is essential for proper synaptic function.

## Huntington Disease: an Overview

Huntington disease (HD) is an autosomal dominant neurodegenerative disease that results in a triad of motor, psychiatric and cognitive symptoms, and has an estimated prevalence of 13.7 per 100,000 in the general population (Fisher and Hayden, 2014). Although HD is considered a brain-wide disease, neuronal degeneration primarily targets spiny projection neurons (SPNs) of the striatum, a brain area essential for regulating voluntary and involuntary movement. HD symptoms typically appear during middle age in most patients; however, disease onset can occur anytime between 1 and 80 years of age. HD is fatal, and life expectancy after diagnosis is estimated to be 15-20 years (Walker, 2007). Symptoms pertaining to the motor system include chorea, dystonia, motor impersistence and motor incoordination. In terms of cognition, HD patients often have problems with tasks involving executive function, such as planning and organizing, and impaired procedural memory (Walker, 2007). Common psychiatric symptoms associated with HD include depression, apathy, aggression and disinhibition, and suicide rates of HD patients are four times higher than that of the general population (Di Maio et al., 1993). Notably, cognitive deficits manifest as many as 20 years earlier than the onset of motor symptoms, and cognitive and behavioral-related issues are reported as the most burdensome for patients (Hamilton et al., 2003; Paulsen, 2011). In HD, these early cognitive abnormalities have mostly been associated with disruption in frontostriatal neural pathways, although multiple other brain areas including the hippocampus also show significant volume loss in the early stages of disease (Rosas et al., 2003). Currently, there is no cure for HD.

Huntington disease is a monogenic disease that is caused by a CAG trinucleotide repeat expansion in exon 1 of the *HTT* gene (also known as the *IT15* gene), which encodes the large, 348 kDa protein huntingtin (HTT) (MacDonald et al., 1993). CAG repeat lengths of 40 or more result in the production of a mutated huntingtin protein (mHTT) while healthy individuals typically have less than 36 repeats. Intermediate CAG repeat lengths of 36-39 result in incomplete penetrance of the HD phenotype (Rubinsztein et al., 1996). Longer CAG repeat expansions are correlated with earlier disease onset (Penney et al., 1997). Wild-type huntingtin (wtHTT; herein used to refer to the non-pathogenic HTT protein) is ubiquitously expressed throughout the body. Within the brain, wtHTT is largely present in nuclei, cell somas, dendrites and terminal buttons, while mHTT has a tendency to accumulate in intranuclear inclusions and dystrophic neurites (DiFiglia et al., 1997). The N-terminal region of wtHTT contains the polyglutamine stretch encoded by the CAG repeat expansion and for this reason has been the most extensively studied portion of the protein, despite accounting for only about 2% of HTT’s structure. In addition, wtHTT contains several HEAT repeats that are important for its numerous protein-protein interactions (Saudou and Humbert, 2016). An essential role of wtHTT is well-documented by the fact that homozygous wtHTT knockout is embryonic lethal at day E8.5 (Duyao et al., 1995; Nasir et al., 1995; Zeitlin et al., 1995). Heterozygous knockout of wtHTT from birth has been shown to result in hyperactivity, deficits in cognitive flexibility and decreased overall volume and neuronal density in the subthalamic nucleus (Nasir et al., 1995). Additionally, wt*HTT* deletion in the forebrain and testis of adult mice results in neurodegeneration, motor impairments and a shortened lifespan (Dragatsis et al., 2000). The essentiality of wtHTT is further supported by its role in numerous fundamental cellular functions due to its extensive number of interaction partners. A few hundred interacting partners had previously been identified using *ex vivo* methods such as yeast two-hybrid and affinity pulldown assays; however, most of these experiments used only small N-terminal fragments of wtHTT, neglecting a large part of its full-length structure (summarized in Table 1 of Harjes and Wanker, 2003). In Shirasaki et al. (2012), the authors used a novel methodological approach to identifying wtHTT interacting partners, which incorporated the use of a high-affinity mass spectrometer. This study was highly successful, identifying 747 candidate proteins from various brain regions that interact with wtHTT. Among the top ranked functional groups for the wtHTT interactors were presynaptic function and postsynaptic function, highlighting a role for wtHTT in synaptic homeostasis. The role that wtHTT plays at both pre- and postsynaptic sites is the main focus of the present review and will be discussed in detail in the following sections.

Wild type HTT has been shown to act as a scaffolding protein, functioning to stabilize intracellular cargo onto molecular motors to streamline fast axonal transport and to assist in anchoring receptors at the plasma membrane (Saudou and Humbert, 2016). As we will discuss throughout this review, wtHTT has been heavily implicated in almost all major facets of synaptic neurotransmission including anterograde and retrograde transport of proteins to/from terminal buttons and dendrites, neurotransmitter release, endocytic vesicle recycling and postsynaptic receptor localization and recycling. Additionally, wtHTT has been shown to influence autophagy, which has been highlighted in recent years as an important regulator of synaptic homeostasis (Vijayan and Verstreken, 2017; Liang and Sigrist, 2018; Nikoletopoulou and Tavernarakis, 2018; Birdsall and Waites, 2019), and synapse-to-nuclear communication via its regulation of transcription factors such as CREB, REST/NRSF and NF-κB (Steffan et al., 2000; Zuccato et al., 2003; Marcora and Kennedy, 2010). In HD, synaptic dysfunction occurs prior to cell death and predicts subsequent neuronal degeneration and symptom onset (Milnerwood and Raymond, 2010; Milnerwood et al., 2010; Parsons and Raymond, 2014; Ravalia et al., 2021). As HD is originally a disease of the synapse, this review will summarize research from the last two decades that provide fundamental evidence for wtHTT as a major regulator of synaptic function and will consider potential and identified consequences of wtHTT depletion at the synapse. It is imperative that we increase our understanding of wtHTT’s function in the developed brain, as many novel therapeutic strategies — discussed in the following section — aim to treat HD by reducing both wtHTT and mHTT expression.

## Targeting the Root Cause: Huntingtin-Lowering Therapeutics

Genetic therapy for HD has shown great promise as a treatment for this crippling disease, with significant recent advancements in the development of both DNA- and RNA-targeting therapies. As HD is a monogenic disease, DNA- and RNA-targeting therapies can target the root cause of the disease itself. Therapies targeting RNA include antisense oligonucleotides (ASOs), RNAi and small molecules, while those targeting DNA include zinc finger nucleases (ZFNs), transcription activator-like effector nucleases (TALENs) and CRISPR-Cas9. Each of the various strategies have specific benefits as well as drawbacks which have been reviewed recently (Tabrizi et al., 2019a). As an example, therapies targeting *HTT* mRNA aim to reduce the production of the mutant protein variant; however, many of these options will also decrease the production of wtHTT. Furthermore, there is evidence that pathogenic exon 1 fragments can result from incomplete splicing at the pre-mRNA level, thereby evading the mRNA-targeting approaches (Sathasivam et al., 2013).

Antisense oligonucleotides target the pre-mRNA stage of mHTT for degradation by RNase H. The delivery and dispersal of ASOs within the central nervous system (CNS) also make these therapeutics particularly desirable. ASOs can penetrate cell membranes without the need for an accompanying viral vector and can be delivered to the brain through intrathecal injections into the spinal cord. The Roche GENERATION HD1 clinical trial using the non-selective ASO tominersen (previously known as HTT_*RX*_ or RG6042) was recently halted early in phase III. Unfortunately, tominersen was no more effective than placebo when administered every 16 weeks, and actually worsened motor and cognitive symptoms when administered every eight weeks (Kwon, 2021). The recent news to terminate this clinical trial is particularly disappointing considering that cerebral spinal fluid (CSF) levels of two established HD biomarkers, mHTT and neurofilament light chain (NfL), showed a dose-dependent decrease after tominersen administration (Tabrizi et al., 2019b).

In terms of selective mHTT lowering therapeutics, Wave Life Sciences initiated their parallel stage I/IIa clinical trials in 2017, PRECISION HD1 and PRECISION HD2, which aim to selectively lower levels of mHTT while leaving wtHTT levels unchanged (Hersch et al., 2017). These selective ASOs are designed to specifically lower mHTT (leaving wtHTT intact) by targeting single nucleotide polymorphisms (SNPs) located exclusively on the mutant allele. Allele-specific ASOs have recently shown promising results in a preclinical study that used humanized HD mice (Southwell et al., 2013); selective mHTT ASOs reduced mHTT expression by approximately 70% while having no effect on wtHTT expression. Furthermore, selective mHTT silencing reduced many of the cognitive and behavioral deficits in these mice (Southwell et al., 2018). Unfortunately, both PRECISION HD1 and PRECISION HD2 trials were recently discontinued as they did not significantly reduce CSF levels of mHTT. Further complicating the SNP-based ASO approach is the fact that HD mutation carriers express different SNPs and some don’t express any heterozygous SNPs at all; therefore this selective strategy is not applicable to the entire HD population (Skotte et al., 2014). Nonetheless, allele-specific ASOs have tremendous promise in the treatment of HD and will continue to be pursued in future clinical trials with similar ASOs that incorporate various chemical modifications designed to improve their efficacy. Another strategy to achieve selective mHTT knockdown is to target the CAG tract itself, although many genes in the human genome also contain consecutive CAG repeats and a number of these genes code for transcription factors. Therefore, targeting the CAG trinucleotide repeat for degradation may result in significant off-target effects. RNAi are another group of RNA-targeting therapies that employ micro RNAs (miRNAs), short interfering RNAs (siRNAs) or short hairpin RNAs (shRNAs) to target mRNA for degradation by RNA-induced silencing complex (RISC) machinery (Aguiar et al., 2017). In contrast to ASOs, RNAi therapies act further downstream and can only target within the intron-lacking mature mRNA. Therefore, RNAi drugs have more limitations in terms of sequence targets (Tabrizi et al., 2019a). As well, these therapies require the use of a viral vector and a more direct injection into the brain. However, a potentially attractive feature of these drugs is their permanence. Whereas patients undergoing clinical trials for ASO therapeutics must receive intrathecal injections every few months, RNAi treatments may only require a single dose. On the other hand, the less reversible nature of these drugs can quickly turn into to a disadvantage if unwanted side-effects are observed. In June 2020, UniQure announced the launch of their phase I/II clinical trial where early manifest HD patients will receive a single intra-striatal administration of AMT-130, a non-selective rAAV5-miRNA (Reilmann et al., 2020). Another type of RNA-targeting therapeutics currently in development for HD are bioavailable small molecules. These drugs are ideal in terms of delivery as they can be taken orally, and positive results have been observed in rodent models of spinal muscular atrophy (SMA) that were treated with small molecules that target and degrade SMN2 RNA (Naryshkin et al., 2014). Gene-editing therapies for the treatment of HD are currently in preclinical development. These drugs target the absolute root cause of HD: the *HTT* (*IT15)* gene. Precise silencing of the mutant allele using gene-editing techniques such as CRISPR-Cas9 would halt m*HTT* transcription at its source; however, gene editing therapies require invasive delivery systems and are largely irreversible. Human clinical trials for DNA-targeting HD therapies are yet to be announced, although initial rodent studies have shown promising results (Yang et al., 2017).

In sum, a plethora of HTT-lowering strategies exist, and all can target the root cause of HD by lowering mHTT expression. However, it is important to note that non-selective HTT silencing is much easier to achieve that allele-selective silencing, and many of the therapeutic options are indeed non-selective therapies that will further reduce wtHTT expression. Therefore, we now turn our attention to the role of wtHTT and the potential consequences of its loss in adulthood.

## Huntingtin and the Synapse

Given the clinical importance and immediate relevance of HTT-lowering therapies for the treatment of HD — many of which are not selective for mHTT over wtHTT — it is of paramount importance to increase our understanding of the function of wtHTT and the consequences of its loss in adulthood. As HD mutation carriers are born with reduced expression of wtHTT, it is also essential to fully understand how wtHTT reduction affects CNS development. In mouse models of HD, one must keep in mind that CAG repeat lengths must be greatly exaggerated before a HD-like phenotype can be observed within the lifespan of a mouse; in that regard, the lack of any obvious consequences of wtHTT loss — particularly at the behavioral level — following a relatively short period of wtHTT reduction is insufficient to conclude that wtHTT loss is well-tolerated. Similarly, while HTT-lowering strategies in the clinic are unlikely to eliminate 100% of the wtHTT and mHTT in the brain, cellular and animal studies that observe deleterious effects only after the complete depletion of wtHTT should not be viewed as lacking physiological relevance; like exaggerated CAG repeat lengths, perhaps complete wtHTT knockdown is the best way to observe the consequences of wtHTT loss within the lifespan of a mouse. In this review, we discuss how wtHTT regulates synaptic function in numerous ways. We chose to focus on wtHTT’s role at the synapse, as synaptic dysfunction is observed prior to cell death and HD behavioral signs, and therefore represents one of the most sensitive measures of disease pathogenesis (Li et al., 2003; Milnerwood and Raymond, 2010; Raymond et al., 2011; Tyebji and Hannan, 2017; Ravalia et al., 2021). In reviewing the multidimensional roles that wtHTT plays at both pre- and postsynaptic sites, we conclude that wtHTT reduction is not well-tolerated at the synaptic level.

## Huntingtin and the Presynapse

**FIGURE 1:**
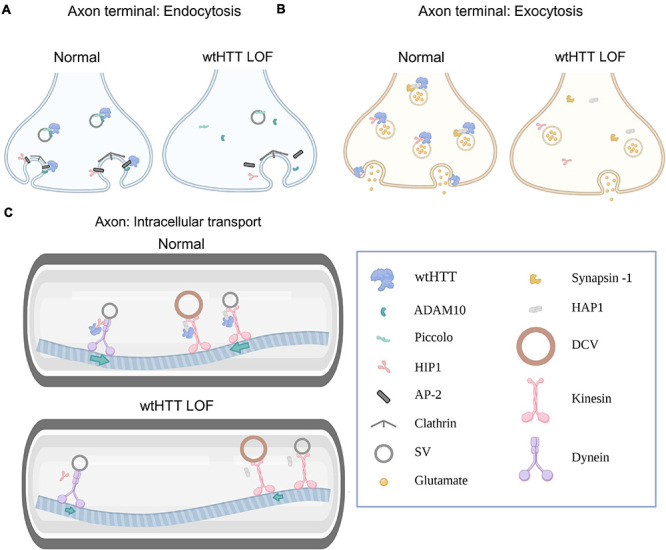
Huntingtin and the presynapse. **(A)** Select examples demonstrating how wtHTT positively regulates endocytosis (left). Through associations with ADAM10 and piccolo, wtHTT regulates SV density at the readily releasable and reserve vesicle pools. Through associations with HIP1, AP-2 and ADAM10, wtHTT regulates clathrin-mediated endocytosis. wtHTT loss impairs endocytosis by disrupting the function of complexes with the aforementioned proteins (right). wtHTT LOF decreases SV density at the readily releasable and reserve vesicle pools and impairs clathrin-mediated endocytosis. **(B)** Select examples demonstrating how wtHTT positively regulates exocytosis. Through its associations with synapsin-1, HAP1 and HIP1, wtHTT can regulate the rate of SV exocytosis and the amount of neurotransmitter release. wtHTT loss disrupts exocytosis (right). **(C)** Select examples demonstrating how wtHTT positively regulates axonal transport (top). Through its associations with HAP1 and HIP1, as well as molecular motors, wtHTT maintains proper anterograde and retrograde transport of cellular cargoes including SVs and DCVs. wtHTT loss impairs axonal transport (bottom). Due to its associations with HAP1 and HIP1, as well as molecular motors, wtHTT loss interferes with anterograde and retrograde transport of cellular cargoes including SVs and DCVs. Abbreviations: LOF, loss of function; ADAM10, A disintegrin and metalloproteinase domain-containing protein 10; HIP1, huntingtin-interacting protein 1; AP-2, adaptor protein complex 2; SV, synaptic vesicle; HAP1, huntingtin-associated protein 1; DCV, dense-core vesicle. Figure created using Biorender.com.

Numerous mechanisms regulating presynaptic neurotransmitter release have been identified to date, though our understanding is far from complete. Presynaptic release is complex and can occur in different modes — including synchronous, asynchronous or spontaneous — depending on whether release is tightly coupled with action potential (AP) firing, exhibits poor temporal coordination with AP firing, or occurs independently of AP firing, respectively. The different modes of presynaptic release are associated with overlapping but distinct underlying mechanisms, and research is ongoing to fully understand how presynaptic release is regulated and maintained at different synapses throughout the healthy brain (Chanaday and Kavalali, 2018). The complexity of presynaptic neurotransmission extends well beyond the successful release of neurotransmitters into the extracellular space. The maintenance of high-fidelity synaptic neurotransmission is a multifaceted process that relies on highly coordinated intracellular mechanisms. For example, presynaptic function relies on the ability to rapidly recycle synaptic vesicles (SVs) (Marx et al., 2015), to ship new SVs from the cell body to the terminal (Okada et al., 1995; Goldstein et al., 2008), to rapidly refill SVs with neurotransmitter (Nakakubo et al., 2020), and to remove damaged proteins from presynaptic sites (Vijayan and Verstreken, 2017). As a result of this complexity, presynaptic function is not only reliant on exocytic release machinery, but also on clathrin-mediated endocytosis, axonal trafficking and autophagy, to name a few. In this section, we will discuss how wtHTT’s role in endocytosis, exocytosis, intracellular transport and autophagy positions wtHTT as a critical mediator of presynaptic neurotransmission. Some of ways in which wtHTT can influence presynaptic function are depicted schematically in Figure 1.

### Huntingtin and the Presynapse: Endocytosis

A finite number of SVs exist at presynaptic release sites. During neural activity, SVs release their contents by fusing with the plasma membrane, and the pool of SVs available within a presynaptic neuron can be rapidly depleted during sustained neural activity. For example, at the CA3-CA1 synapse in the hippocampus, it is estimated that approximately 30 seconds of neural activity at a physiologically relevant firing rate of four hertz is sufficient to completely deplete the presynaptic supply of glutamate SVs (Marx et al., 2015). To maintain neurotransmission in the face of sustained neural activity, numerous mechanisms are in place to help ensure a rapid recovery and replenishment of the SV pool. Often, the rate of SV fusion with the membrane during neural activity far exceeds the rate at which new SVs can be delivered via anterograde transport from the cell body; thus, an essential method of SV replenishment during activity is through local clathrin-mediated endocytosis at the terminal itself.

A role for wtHTT in presynaptic neurotransmission was suggested by its association with SVs (DiFiglia et al., 1995; Yao et al., 2014). Recently, it was demonstrated that a key wtHTT interactor, ADAM10, is heavily involved in presynaptic homeostasis. ADAM10 is a transmembrane protease that is well-established as an alpha-secretase which cleaves amyloid precursor protein (APP) in a non-amyloidogenic fashion (Kuhn et al., 2010). ADAM10 localizes presynaptically with SVs, suggesting a role in presynaptic regulation (Lundgren et al., 2020). While Alzheimer disease (AD) is associated with reduced ADAM10 levels (Kuhn et al., 2010), thereby resulting in excess amyloidogenic processing of amyloid precursor protein, ADAM10 is hyperactive in the HD brain at both pre- and postsynaptic localizations (Cozzolino et al., 2021). Using immunoprecipitation followed by mass spectrometry and gene ontology analysis, it was found that the ADAM10 interactome exhibits substantial overlap with the wtHTT interactome; many of the shared interactors were identified as proteins essential for presynaptic function, thereby highlighting a putative functional role for both wtHTT and ADAM10 at the presynaptic active zone. For example, both ADAM10 (Cozzolino et al., 2021) and wtHTT (Yao et al., 2014) bind to piccolo, a large cytomatrix protein that is critical for SV maintenance and efficient SV recycling (Ackermann et al., 2019). Hyperactive ADAM10, which can be induced either by pathogenic polyQ expansion of HTT or by wtHTT loss (Lo Sardo et al., 2012), disrupts the ADAM10/piccolo complex, resulting in depleted SVs at the readily releasable and reserve vesicle pools. Restoring ADAM10 activity to control levels in HD mice, achieved by crossing R6/2 mice with heterozygous conditional ADAM10 knockout mice (CaMKIIα-Cre:Adam10^Flox/+^), restored the ADAM10/piccolo interaction and replenished SV stores (Cozzolino et al., 2021). Thus, wtHTT loss may have detrimental effects on presynaptic homeostasis by interrupting the HTT/ADAM10/piccolo complex. In addition to complexing with ADAM10/piccolo and other presynaptic regulatory proteins including bassoon (Yao et al., 2014), wtHTT and ADAM10 both interact with the clathrin adaptor protein AP-2 (Borgonovo et al., 2013; Marcello et al., 2013), and wtHTT serves as a docking protein that helps recruit AP-2 to the membrane. Interestingly, polyQ expansion of HTT results in a loss of wtHTT’s docking function, thereby reducing AP-2 presence at the membrane and impairing clathrin-mediated endocytosis (Borgonovo et al., 2013).

Wild type HTT’s interactions with huntingtin interacting protein 1 (HIP1) also position wtHTT to influence presynaptic transmission. When the wtHTT/HIP1 interaction was first described, it was recognized that disrupting this interaction could negatively impact the integrity of the cytoskeleton (Kalchman et al., 1997). A few years later, HIP1 was implicated in endocytosis through interactions with both clathrin and the AP-2 adaptor complex (Metzler et al., 2001; Mishra et al., 2001; Waelter et al., 2001). Consistent with a role in synaptic vesicle recycling through endocytosis, HIP1 knockout mice exhibit a slower recovery from synaptic depression (Parker et al., 2007).

Together, the aforementioned studies suggest that wtHTT loss of function (LOF) may disrupt presynaptic homeostasis by impairing clathrin-mediated SV recycling. A direct role of wtHTT in SV endocytosis was demonstrated recently when self-deliverable, cholesterol-conjugated siRNAs were used to knock down wtHTT expression in cultured neurons (McAdam et al., 2020). In this study, wtHTT levels were reduced to approximately 20% of that observed in control neurons, and the fluorescent reporter synaptophysin-pHluorin (syp-pH) was used to quantify the rate of SV recycling. Syp-pH fluorescence is quenched when in the acidic environment inside SVs, and SV exocytosis during neural activity increases syp-pH fluorescence. In striatal cultures with reduced wtHTT expression, the rate of SV recovery — quantified by the decay of the evoked syp-pH transient — was slower in striatal cultures with reduced wtHTT expression. SV recycling was also impaired in cells cultured from knock-in HD mice and this deficit could be fully rescued by the overexpression of wtHTT. Together, these data demonstrate a clear role for wtHTT as a positive regulator of SV recycling. Interestingly, the recycling rate was unaffected in cultured hippocampal neurons following wtHTT knockdown, suggesting that the consequences of wtHTT reduction on synaptic function varies in a region-dependent manner (McAdam et al., 2020).

In sum, wtHTT interacts with key presynaptic proteins that regulate SV endocytosis, and wtHTT LOF slows the rate of SV recycling following a period of evoked neural activity. It will be of interest for future studies to further pinpoint the mechanisms underlying wtHTT’s role in SV recycling and to determine how much wtHTT reduction in the adult brain can be safely tolerated before SV replenishment rates are negatively impacted.

### Huntingtin and the Presynapse: Exocytosis

The effects of wtHTT loss on SV exocytosis are not fully understood, though mHTT expression was shown to inhibit exocytosis by depleting complexin II (Edwardson et al., 2003). Many of the proteins that complex together with wtHTT have been shown to play a clear role in exocytic neurotransmitter release, and wtHTT lowering can interfere with the normal functions of these protein complexes. For example, the wtHTT binding partner huntingtin associated protein 1 (HAP1) (Li et al., 1995), interacts with the presynaptic protein synapsin-I, and HAP1 depletion was shown to reduce both the rate of SV exocytosis and the amount of evoked glutamate release in excitatory neurons (Mackenzie et al., 2016). Similarly, HIP1 knockout increases the paired-pulse ratio measured at hippocampal CA3-CA1 synapses, indicative of a decrease in neurotransmitter release probability (Parker et al., 2007). Another major wtHTT interactor, huntingtin interacting protein 14 (HIP14), has also been shown to facilitate presynaptic neurotransmitter release. HIP14 is a palmitoyl acyltransferase that palmitoylates target substrates including a variety of presynaptic proteins such as cysteine string protein and SNAP25. wtHTT is a positive regulator of HIP14 (Huang et al., 2011), and HIP14 depletion impairs activity-dependent SV exocytosis at the neuromuscular junction in *Drosophila* (Ohyama et al., 2007) and reduces electrophysiological measures of release probability at glutamatergic synapses within the striatum (Milnerwood et al., 2013). When HIP14 knockdown is initiated in adulthood, reduced release probability and mEPSC frequency is observed in SPNs, and the mice exhibit motor deficits and increased anxiety-like behaviors (Sanders et al., 2016). In HeLa cells, wtHTT itself is directly involved in secretory vesicle fusion with the plasma membrane during exocytosis (Brandstaetter et al., 2014). Presynaptic wtHTT expression has also been implicated in long-term synaptic plasticity, as long-term facilitation of the sensory-to-motor neuron synapse was impaired when the Aplysia wtHTT homolog was silenced by ASO injection into the presynaptic sensory neuron (Choi et al., 2014). While additional questions remain regarding wtHTT’s precise role in exocytosis, multiple lines of evidence indicate that wtHTT and its interactome are essential components of the presynaptic machinery regulating exocytic neurotransmitter release.

### Huntingtin and the Presynapse: Axonal Transport

One particularly well-acknowledged function of wtHTT is its role intracellular transport. The wtHTT/HAP1 complex influences intracellular trafficking by forming larger complexes with kinesin and dynein molecular motors, which are responsible for anterograde (away from the cell body) and retrograde (toward the cell body) transport of various molecular cargoes, respectively (Engelender et al., 1997; Li et al., 1998; McGuire et al., 2006; Saudou and Humbert, 2016; Vitet et al., 2020). Both pathogenic polyQ expansion of HTT (Li et al., 1995) and wtHTT loss have been shown to impair axonal trafficking by interfering with the known functions of the HTT/HAP1 complex (Gunawardena et al., 2003; Gauthier et al., 2004; Zala et al., 2013). Perhaps the most acknowledged functional outcome of wtHTT’s trafficking role is in the anterograde delivery of BDNF to the striatum from presynaptic cortical neurons. The striatum produces low amounts of this trophic factor on its own and striatal neurons require BDNF delivery from cortical terminals for long-term survival (Baquet et al., 2004). Through associations with HAP1 and the p150^*Glued*^ subunit of dynactin, an essential co-factor of the dynein molecular motor, wtHTT facilitates both anterograde and retrograde BDNF transport along microtubules, and loss of wtHTT is sufficient to slow BDNF transport (Gauthier et al., 2004). In addition to facilitating BDNF transport, wtHTT also enhances BDNF synthesis by sequestering REST, a transcription factor that normally acts in the nucleus to silence BDNF expression (Zuccato et al., 2001, 2003). Thus, by increasing synthesis and accelerating the intracellular transport of BDNF, wtHTT plays a critical role in delivering presynaptic trophic support to the striatum. BDNF is important not only for survival but is also essential for synaptic plasticity (Harward et al., 2016), and BDNF deficiencies may in fact underlie synaptic plasticity deficits observed in mouse models of HD (Lynch et al., 2007; Simmons et al., 2009). Interestingly, synaptic plasticity deficits occur earlier and are more severe in homozygous knock-in HD mice (which completely lack wtHTT) compared to heterozygous knock-in HD mice, although it is not known whether this accelerated plasticity deficit results from a higher expression of mHTT or the lack of wtHTT in the homozygous HD mice (Quirion and Parsons, 2019).

Numerous post-translational modifications (PTMs) of wtHTT have been shown to be essential to its role in vesicular transport. For example, serine 421 (S421) on wtHTT has been identified as an important phosphorylation site that mediates axonal trafficking; phosphorylation of wtHTT at S421 recruits kinesin-1 and promotes anterograde transport of BDNF-containing vesicles whereas S421 dephosphorylation favors retrograde transport following kinesin-1 detachment (Colin et al., 2008). On the other hand, dephosphorylation at S1181 and S1201 strengthens molecular motor attachment to microtubules and enhances the transport of BDNF (Ben et al., 2013). In addition to phosphorylation, the arginine methyltransferase PRMT6 was recently shown to methylate arginine R118 of wtHTT, increasing wtHTT’s association with vesicles and facilitating vesicular trafficking (Migazzi et al., 2021). Knocking down PRMT6 or transfecting neurons with a methylation-resistant wtHTT (R118K) reduced both the number and speed of vesicles traveling in the anterograde direction. Increasing methylation was able to rescue axonal transport deficits in mHTT-expressing neurons and was protective in a fly model of HD, highlighting methylation and the restoration of wtHTT’s trafficking function as a potential therapeutic strategy for HD (Migazzi et al., 2021).

Impaired axonal trafficking can have profound functional consequences that extend well beyond the aforementioned reduction in BDNF delivery to the striatum. Efficient trafficking of a variety of cargo both to and from synaptic compartments is essential for the maintenance of synaptic homeostasis. For example, wtHTT loss interferes with the delivery of large dense core vesicles (DCV), which carry neurotrophins and neuropeptides, to release sites (Weiss and Littleton, 2016; Bulgari et al., 2017). Growing evidence supports a key role of APP in regulating synaptic structure and function (Priller et al., 2006; Tyan et al., 2012; Müller et al., 2017), and wtHTT also facilitates the transport of APP to the presynapse (Colin et al., 2008; Her and Goldstein, 2008; Bruyère et al., 2020). Either silencing wtHTT (Her and Goldstein, 2008) or preventing wtHTT phosphorylation at S421 (Bruyère et al., 2020) impairs APP axonal transport. Thus, wtHTT dephosphorylation reduces the amount of APP at presynaptic compartments (Bruyère et al., 2020). By reducing APP at presynaptic sites, wtHTT dephosphorylation increases synapse density in CA1 stratum radiatum. As well, the excessive synaptic connectivity induced by wtHTT dephosphorylation can be restored by APP overexpression (Bruyère et al., 2020). The finding that wtHTT dephosphorylation increased the volume of the cortex and hippocampus but not the striatum suggests that regional sensitivities to the consequence of wtHTT LOF do not necessarily mimic the known regional sensitivities to mHTT toxicity (Bruyère et al., 2020).

Anterograde axonal transport of SV precursors (SVPs) is required to bring the proper release machinery to the presynapse. Newly synthesized SVP delivery works together with endocytosis, albeit at a slower rate, to contribute to the maintenance of presynaptic SV supply (Guedes-Dias and Holzbaur, 2019). SVPs are vesicles containing essential presynaptic proteins that are required to fill, dock and release SVs at terminal buttons. These SVPs travel from the cell body to axon terminals by kinesin-mediated anterograde transport. Fluorescence recovery after photobleaching demonstrates that HAP1 facilitates the axonal trafficking of synapsin-I-positive SVs to axon terminals. In neurons cultured from HAP1 knockout mice, the transport rate of synapsin-I was reduced by approximately 50% (Mackenzie et al., 2016). While the authors did not investigate wtHTT in this study, it is conceivable that wtHTT LOF may produce similar effects by interfering with the efficiency of the HTT/HAP1 complex. Indeed, in *Drosophila*, knocking out the *Drosophila* homolog of wtHTT was found to slow axonal trafficking of synaptotagmin-containing SVs (Zala et al., 2013). More recent evidence demonstrates that wtHTT moves along the axon together with Rab4^+^ SVs that also contain synaptic SNARE proteins synaptotagmin and synaptobrevin (White et al., 2020). Rab4 is a Rab GTPase that plays a key role synaptic homeostasis by controlling the recycling and degradation of synaptic vesicles (Dey et al., 2017). The bidirectional movement of these Rab4^+^ vesicles was mediated by interactions with HIP1, rather than HAP1, and the molecular motors kinesin-1 and dynein. RNAi-mediated wtHTT reduction reduced the axonal mobility of Rab4^+^ vesicles (White et al., 2020). Together, wtHTT appears to facilitate the axonal trafficking of a variety of cargos, many of which have essential roles in presynaptic fidelity and SV maintenance.

### HTT and the Presynapse: Autophagy

In recent years, it has become clear that autophagy is more than a simple housekeeping process that rids the cell of unwanted materials. In fact, autophagy is being increasingly recognized as a major contributor to synaptic function, which has been recently reviewed elsewhere and will not be extensively covered in the present review (Vijayan and Verstreken, 2017; Liang and Sigrist, 2018; Nikoletopoulou and Tavernarakis, 2018; Birdsall and Waites, 2019). Synapses are particularly sensitive to proteostatic disruption, and at presynaptic sites, autophagy is not only essential for removing defective proteins but can also influence neurotransmitter release. For example, enhancing presynaptic autophagy reduced the size of dopamine (DA) terminals, the number of synaptic vesicles found at DA terminals and the magnitude of evoked DA release (Hernandez et al., 2012). In contrast, when Atg7 — an essential protein for autophagic vesicle formation — was deleted in DA neurons, the opposite effects were observed; in these autophagy-deficient mice, DA axon profiles were larger, evoked DA release was enhanced, and presynaptic recovery following evoked DA release was accelerated (Hernandez et al., 2012). In HD, mHTT can increase the number of autophagosomes by sequestering mTOR, which normally functions to inhibit phagophore formation (Ravikumar et al., 2004). However, these autophagosomes that accumulate in HD cells are largely devoid of cargo due to cargo recognition failure (Martinez-Vicente et al., 2010). wtHTT has been shown to be an important scaffold protein that facilitates selective autophagy — the removal of specific cytoplasmic materials rather than bulk degradation. Through physical interactions with the cargo adaptor p62 and the autophagy initiation kinase ULK1 proteins, wtHTT plays an essential role in both selective autophagy cargo recognition and autophagy initiation (Rui et al., 2015). Post-translational myristylation of wtHTT at Gly553 promotes the formation of autophagosomes (Martin et al., 2014, 2015), and autophagy dysfunction was observed in wtHTT conditional knockout mice where wtHTT loss was driven by the nestin promoter (Ochaba et al., 2014). Consistent with a wtHTT role in intracellular trafficking, both wtHTT and HAP1 also contribute to autophagosome content degradation by facilitating autophagosome transport to the lysosome. wtHTT and HAP1 were identified in autosome-enriched fractions, and siRNA-mediated wtHTT knockdown decreased the number and speed of autophagosomes traveling in the retrograde direction while increasing the number of stationary autophagosomes (Wong and Holzbaur, 2014). In sum, wtHTT serves as a positive regulator of autophagy, autophagic deficits are observed following wtHTT loss, and autophagy is now recognized to play an essential role in presynaptic neurotransmission and synaptic homeostasis. It will be of interest for future studies to determine the precise extent of synaptic dysfunction caused by wtHTT loss, and how much of that impairment can be attributed to defective autophagic mechanisms.

## Huntingtin and the Postsynapse

**FIGURE 2:**
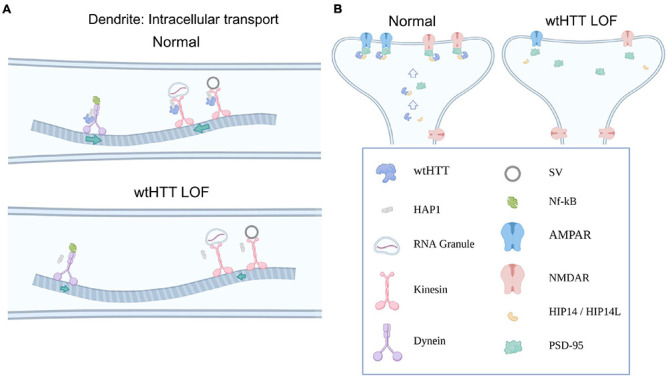
Huntingtin and the postsynapse. **(A)** Select examples demonstrating how wtHTT positively regulates dendritic transport (top). Through its associations with HAP1, as well as molecular motors, wtHTT regulates anterograde and retrograde transport of synaptic receptors and other cargo in dendrites (left). wtHTT LOF disrupts this transport and slows delivery of synaptic cargo to their respective anterograde or retrograde targets (bottom). **(B)** Select examples demonstrating how wtHTT positively regulates receptor localization (left). Through its associations with HIP14 and PSD-95, wtHTT regulates synaptic receptor stabilization at the PSD in a healthy postsynaptic neuron. wtHTT LOF disrupts postsynaptic protein clustering and receptor localization (right). Abbreviations: LOF, loss of function; HAP1, huntingtin-associated protein 1; SV, synaptic vesicle; NF-κB, nuclear factor kappa B; AMPAR, α-Amino-3-hydroxy-5-methyl-4-isoxazolepropionic acid receptor; NMDAR, N-Methyl-D-aspartate receptor; HIP14/HIP14L, huntingtin-interacting protein 14/huntingtin-interacting protein 14-like; PSD-95, postsynaptic density protein 95. Figure created using Biorender.com.

In addition to the myriad ways in which wtHTT positively influences presynaptic neurotransmission as discussed above, ample evidence exists to suggest that wtHTT is equally as important to proper postsynaptic function. In this section, we discuss how wtHTT is involved in the bidirectional transport of essential synaptic cargoes between the soma and dendritic tree, postsynaptic receptor clustering and subcellular localization, as well as spine stabilization and synaptic plasticity. Some of ways in which wtHTT can influence postsynaptic function are depicted schematically in Figure 2.

### Huntingtin and the Postsynapse: Dendritic Transport

Like the presynapse, bidirectional transport of a variety of cargoes between the cell body and postsynaptic sites is critical for proper synapse formation and function. The wtHTT/HAP1 complex, through an interaction with kinesin family motor protein 5 (KIF5), assists the delivery of GABA_*A*_ receptors to postsynaptic sites at inhibitory synapses (Twelvetrees et al., 2010). Disrupting the HAP1/KIF5 interaction reduced the surface expression of GABA_*A*_ receptors and decreased the amplitude of miniature inhibitory postsynaptic currents (mIPSCs). Interestingly, wtHTT/HAP1/KIF5 protein complexes also deliver AMPA receptors to postsynaptic sites at excitatory synapses. In cultured neurons, wtHTT increased the strength of the interaction between HAP1 and KIF5 motors, promoting the transport GluA2-containing vesicles along microtubules to reach postsynaptic sites. Interfering with the protein complex, either through mHTT presence or by knocking down HAP1 or KIF5, reduced AMPA receptor trafficking and decreased mEPSC amplitude. Overexpressing wtHTT had the opposite effect, increasing mEPSC amplitude at excitatory synapses (Mandal et al., 2011). In addition to delivering GABA and glutamate receptors to postsynaptic sites, wtHTT also promotes soma-to-dendrite transport of RNA granules, which allows localized protein translation to occur at dendric sites (Savas et al., 2010). For example, via interactions with HAP1 and KIF5 motors, wtHTT helps bring β-actin mRNA to dendrites. Local dendritic translation of β-actin is important for dendritic growth and plasticity (Eom et al., 2003), and it was shown that shRNA-mediated knockdown of either wtHTT, HAP1 or KIF5 reduced the amount of β-actin mRNA in dendrites (Ma et al., 2011).

Wild type HTT also colocalizes with the BDNF receptor TrkB at postsynaptic sites. As mentioned above, wtHTT depletion can negatively impact BDNF production and its anterograde delivery to synaptic sites (Zuccato et al., 2001; Gauthier et al., 2004). At the postsynapse, siRNA-mediated wtHTT knockdown was shown to impair the anterograde transport of BDNF TrkB receptors to striatal dendrites (Liot et al., 2013), in agreement with reports of reduced TrkB expression in the context of HD (Ginés et al., 2006). Once activated by BDNF, TrkB receptors internalize and are transported to the cell body where they can exert pro-growth and survival effects in the postsynaptic neuron (Zheng et al., 2008), and wtHTT reduction also slows TrkB receptor retrograde transport in dendrites (Liot et al., 2013). Furthermore, it was recently demonstrated that autophagosomes are responsible for the retrograde transport of BDNF-activated TrkB receptors via associations between the p150^*Glued*^ subunit of dynactin and the adaptor protein AP-2. The AP-2-mediated retrograde transport of BDNF/TrkB autophagosomes was shown to bring active TrkB to the soma where it promoted neuronal complexity and protected against neurodegeneration (Kononenko et al., 2017). As discussed above, wtHTT loss impairs autophagosome transport (Wong and Holzbaur, 2014), which may help explain why TrkB retrograde transport is reduced following wtHTT knockdown (Liot et al., 2013). Thus, in addition to decreasing BDNF production and axonal trafficking to presynaptic cortico-striatal terminals (Zuccato et al., 2001; Gauthier et al., 2004), wtHTT loss can also interfere with the delivery of TrkB receptors to the postsynapse, as well as the transport of activated TrkB receptors from the dendrite to the soma, thereby interfering with BDNF/TrkB’s normal pro-survival and pro-growth effects at multiple levels. Similarly, the wtHTT/HAP1 complex has also been implicated in TrkA internalization and trafficking, which is required to promote neurite outgrowth (Rong et al., 2006). In agreement with a role of wtHTT in promoting postsynaptic growth and survival, wtHTT knockdown in adulthood reduces the survival of newly born neurons in the dentate gyrus and diminishes dendritic complexity (Pla et al., 2013).

Transcriptional dysregulation is a major component of HD pathogenesis (Kumar et al., 2014), and previous literature suggests that the transcriptional dysregulation observed in HD may be partially recapitulated by wtHTT reduction. For example, NF-κB is a ubiquitous transcription factor that controls the expression of numerous genes with wide ranging functions including cell survival and synaptic plasticity among many others (Kaltschmidt and Kaltschmidt, 2015). NF-κB translocates to the nucleus to activate target genes, and this translocation occurs in an activity-dependent manner that relies on retrograde transport via dynein motors (Mikenberg et al., 2007). In the postsynaptic density, wtHTT co-localizes with NF-κB, and in cultured neurons from conditional wtHTT knockout mice — mice lacking wtHTT in cortical neurons — the activity-dependent transport of NF-κB out of dendritic spines was slowed two-fold. Similarly, wtHTT loss reduced NF-κB activity in the nucleus, demonstrating that wtHTT LOF impairs the movement of NF-κB from synaptic sites to the nucleus, thereby inhibiting its actions on target genes (Marcora and Kennedy, 2010). While NF-κB has myriad effects on gene expression when in the nucleus, it is thought that the ongoing synaptic activity that drives the tonic level of nuclear NF-κB is neuroprotective (Bhakar et al., 2002; Fridmacher et al., 2003). Thus, wtHTT reduction may decrease neuronal survival by reducing the activity-dependent dendrite-to-nucleus transport of this ubiquitous transcription factor. Overall, wtHTT plays a clear role in both axonal and dendritic transport, and many of the identified cargoes trafficked with wtHTT’s assistance play essential roles in both pre- and postsynaptic function. As more and more huntingtin-lowering strategies enter the clinic, we desperately need to increase our understanding of how wtHTT loss in adulthood impacts the delivery efficiency of essential proteins to pre- and postsynaptic sites.

### Huntingtin and the Postsynapse: Receptor Localization and Cellular Toxicity

Through interactions with palmitoyl acyltransferases HIP14 and HIP14-like (HIP-14L), wtHTT acts as a positive modulator of palmitoylation, a PTM known to affect the subcellular localization and clustering of numerous synaptic proteins. For example, palmitoylation of PSD-95 targets this important postsynaptic scaffold to synaptic sites, and thereby regulates glutamate receptor clustering and activity-dependent synaptic plasticity (Craven et al., 1999; El-Husseini et al., 2002). Direct palmitoylation of AMPA and NMDA receptor subunits also regulates their trafficking and localization at synaptic sites (Hayashi et al., 2005, 2009). wtHTT has been identified as a positive modulator of HIP14, as either mHTT presence or wtHTT loss can reduce the enzymatic activity of HIP14 (Huang et al., 2011). Disrupting the function of these PATs has serious consequences at both the synaptic and behavioral levels. Behaviorally, HIP14 knockout mice, as well as HIP14L knockout mice, recapitulate many of the motor and cognitive impairments observed in HD mouse models (Singaraja et al., 2011; Milnerwood et al., 2013; Sutton et al., 2013). At the postsynaptic level, HIP14 knockout mice exhibit enhanced excitability of striatal SPNs, as well as decreased spine density and impaired synaptic plasticity in the hippocampus; the latter likely contributing to the observed spatial memory deficits in HIP14 knockout mice (Milnerwood et al., 2013). Consistent with a role for wtHTT as a positive modulator of synaptic protein palmitoylation, wtHTT overexpression was also shown to increase the palmitoylation and clustering of PSD-95 at synaptic sites, while ASO-mediated knockdown of wtHTT reduced PSD-95 cluster size (Parsons et al., 2014). Recently, it was shown that HIP14L, which also interacts with HTT (Sutton et al., 2013), palmitoylates cluster II of the GluN2B NMDA receptor subunit, and reducing cluster II palmitoylation on GluN2B increases GluN2B-containing NMDA receptor presence at extrasynaptic sites (Kang et al., 2019). Based on structural similarities to HIP14, it is likely that wtHTT also enhances the enzymatic activity of HIP14L (Sanders et al., 2014), as it does for HIP14 (Huang et al., 2011). As one of the earliest identified synaptic abnormalities to occur in HD mice is the overexpression of GluN2B-containing extrasynaptic NMDARs that are preferentially coupled to cell-death pathways (Okamoto et al., 2009; Milnerwood and Raymond, 2010; Milnerwood et al., 2010; Parsons and Raymond, 2014), reduced GluN2B cluster II palmitoylation and subsequent NMDAR mislocalization may play a key role in early HD synaptic dysfunction. Together, these data suggest that by facilitating palmitoylation, wtHTT plays an important role in synaptic protein organization, particularly at the postsynaptic density.

Through interaction with PSD-95, wtHTT forms a complex with NMDA and kainate receptors, and decreasing the interaction between HTT and PSD-95 can sensitize NMDARs (Sun et al., 2001). Thus, it is suggested that wtHTT normally functions to limit NMDAR toxicity through strong interactions with PSD-95. Indeed, overexpression of wtHTT protects against excitotoxicity (Leavitt et al., 2006) and can even limit the toxicity caused by mHTT presence (Leavitt et al., 2001). Furthermore, wtHTT also interacts with caspase-3 and inhibits this pro-apoptotic executioner caspase. siRNA-mediated wtHTT knockdown increases the level of active caspase-3, and hippocampal levels of caspase-3 are significantly elevated in wtHTT-depleted cells in chimeric mice that contain populations of wtHTT-lacking neurons (Zhang et al., 2006). In addition, HAP1 and wtHTT interact with IP3 receptors, which module calcium release from internal stores, to form a ternary complex. While it remains to be determined how wtHTT loss affects the function of these IP3 receptors, it was shown that mHTT presence increases the sensitivity of IP3 receptors to IP3, thereby contributing to toxicity through the excessive release of intracellular calcium stores (Tang et al., 2003). Thus, while receptor mislocalization and enhanced excitotoxicity are well-accepted as major pathogenic mechanisms in the neurobiology of HD, there are numerous routes through which wtHTT reduction could result in similar outcomes.

In addition to palmitoylation discussed above, both wtHTT and mHTT are subject to numerous other PTMs including proteolysis, phosphorylation and myristoylation, to name a few (Ehrnhoefer et al., 2011; Saudou and Humbert, 2016). Considering the large size of the HTT protein and that most research has focused on its N-terminal region, it is unlikely that all HTT PTMs have been characterized. However, many PTMs discovered thus far have direct implications in the toxicity of HTT, some of which can even turn non-pathogenic wtHTT into a toxic protein. After translation, HTT is subject to various proteolysis events by proteases such as caspases and calpains. Notably, caspase cleavage of mHTT, particularly at the caspase-6 cleavage site, is an essential contributor to mHTT toxicity (Wellington et al., 2000; Graham et al., 2006), and the presence of N-terminal mHTT fragments alone is sufficient to produce a robust HD-like phenotype in animal models (Mangiarini et al., 1996). Interestingly, there is evidence to suggest that the C-terminal fragments produced by HTT cleavage — fragments that do not contain the mutant-defining polyQ stretch — can cause endoplasmic reticulum stress and cellular toxicity. This C-terminal fragment toxicity was due to the interaction with and inactivation of dynamin-1, which inhibited endocytosis at the plasma membrane and increased endoplasmic reticulum vacuolation (El-Daher et al., 2015). The surprising finding that protease-mediated cleavage may convert the protective wtHTT protein into a toxic protein fragment highlights the need to further understand the toxic and protective mechanisms of relevant HTT fragments, not just those at the N-terminal region containing the glutamine repeat. Phosphorylation is another PTM that can directly influence HTT’s contribution to cellular toxicity. For example, wtHTT is phosphorylated at S1181 and S1201 by cyclin-dependant kinase 5, and this PTM directly impacts striatal neuron survival. While phosphorylation at these sites was found to protect against mHTT toxicity, dephosphorylation at S1181 and S1201 in wtHTT increased cell death in striatal neurons (Anne et al., 2007). Recently, a single nucleotide polymorphism (SNP) was identified that inhibits post translational myristoylation of wtHTT, which results in impaired cell health due to caspase cleavage at D513 (Martin et al., 2018). Thus, in addition to the effects of PTMs on wtHTT’s role in vesicular trafficking described earlier in this review, numerous PTMs have been identified that can abolish the protective properties of wtHTT or even convert it to a toxic protein itself.

### Huntingtin and the Postsynapse: Synaptic Stability and Plasticity

In addition to the previously discussed role of ADAM10 in presynaptic SV regulation, this metalloproteinase is also found in dendrites and spines and is known to play a major role in postsynaptic function. wtHTT inhibits ADAM10, and hyperactive ADAM10 can result from mHTT presence or from wtHTT loss (Lo Sardo et al., 2012). Hyperactive ADAM10 leads to excessive cleavage of N-cadherin which decreases synapse stability by reducing post- and presynaptic membrane adhesion. In the context of HD, inhibiting ADAM10 can partially rescue the reduced EPSC frequency recorded from SPNs in R6/2 and zQ175 mice (Vezzoli et al., 2019). As wtHTT loss also increases ADAM10 activity (Lo Sardo et al., 2012), it can be predicted that wtHTT loss would have similar effects on N-cadherin processing and synaptic dysregulation. Interestingly, when wtHTT is knocked out in developing cortical neurons, the excessive synaptic connections that are formed early cannot be maintained (McKinstry et al., 2014). While the McKinstry 2014 study did not investigate ADAM10 activity, their finding of decreased spine stability following wtHTT loss is consistent with wtHTT’s regulatory role over ADAM10 and the cleavage of its substrates like N-cadherin.

Synaptic plasticity deficits have been observed in numerous mouse models of HD (Usdin et al., 1999; Murphy et al., 2000; Lynch et al., 2007; Simmons et al., 2009; Brito et al., 2014; Giralt et al., 2017; Quirion and Parsons, 2019) as well as in HD patients (Orth et al., 2010). While it is unknown to what extent wtHTT LOF contributes to the observed plasticity deficits in the context of HD, there are numerous mechanisms by which wtHTT reduction could negatively impact synaptic plasticity in the brain. For example, hippocampal long-term potentiation (LTP) is heavily reliant on BDNF (Lu et al., 2014), and we have already discussed how wtHTT upregulates the synthesis and trafficking of this essential neurotrophin (Zuccato et al., 2001; Gauthier et al., 2004). Therefore, it can be speculated that a decrease in BDNF synthesis and/or delivery to synaptic sites following wtHTT loss may impair hippocampal LTP. In HD mice, exogenous BDNF application is sufficient to restore LTP to control levels (Lynch et al., 2007). CREB binding protein (CBP), a histone acetyltransferase and CREB co-activator that helps regulate the expression of a number of plasticity-related genes, is a known wtHTT interactor and its transcriptional activity is disrupted in HD mouse models (Steffan et al., 2000; Cong et al., 2005; Jiang et al., 2006). Furthermore, by reducing the clustering of PSD-95 at postsynaptic sites (Parsons et al., 2014), wtHTT loss may reduce the number of trapping slots available for AMPA receptor insertion during LTP (Opazo et al., 2012). It is also conceivable that wtHTT loss can impact long-term depression (LTD) by interfering with the normal function of the wtHTT/HIP1 complex. In addition to a role for this complex in the presynapse as discussed above, HIP1 is also known to be particularly enriched in dendrites and at postsynaptic sites (Metzler et al., 2003; Okano et al., 2003; Yao et al., 2006), where it colocalizes and interacts with GluA1. Activity-dependent AMPA receptor internalization is absent in HIP1 knockout mice, with attenuated internalization observed in heterozygous mice (Metzler et al., 2003). Excess unbound HIP1, which may arise either through mHTT presence (Kalchman et al., 1997) or loss of wtHTT, increases the activation of proapoptotic caspases including caspase-3 and caspase-8 (Hackam et al., 2000; Gervais et al., 2002; Zhang et al., 2006). In addition to its well-accepted role as an executioner caspase, transient caspase-3 activation is required for NMDA receptor-dependent LTD (Li et al., 2010). In sum, while largely speculative at present, numerous possibilities exist in which a reduction in wtHTT can negatively impact synaptic plasticity. Indeed, it has been demonstrated that postsynaptic expression of the wtHTT homolog in Aplysia is required for long-term facilitation of excitatory responses at the sensory-to-motor neuron synapse (Choi et al., 2014). More recently, shRNA-mediated wtHTT knockdown in the hippocampus was found to prevent novel location learning (Shen et al., 2019). It is important for future studies to determine how wtHTT reductions impact different forms of synaptic plasticity in different brain regions, as cognitive disturbances can emerge early in HD-mutation carriers and are often reported to be the most debilitating aspect of the disease (Paulsen, 2011).

## HTT Loss *in vivo* During Development

It is indisputable that wtHTT is essential for development, as its knockout is embryonic lethal (Duyao et al., 1995; Nasir et al., 1995; Zeitlin et al., 1995). Furthermore, heterozygous wtHTT knockout mice exhibit motor and cognitive deficits, as well as substantial neuronal loss (up to 50%) in the globus pallidus and the subthalamic nucleus (Nasir et al., 1995; O’Kusky et al., 1999). However, no cell death is observed in the striatum of these heterozygous mice, again demonstrating that the regional sensitivities to wtHTT loss do not necessarily mirror the regional sensitivities of mHTT toxicity.

Conditional wtHTT models also clearly demonstrate that wtHTT is necessary for proper synapse development, function, and stability. Interestingly, early hyperexcitability that cannot be maintained has been reported in HD (Joshi et al., 2009). In the YAC128 mouse model of HD, increased glutamate release and excitatory currents are observed in the striatum at one month of age — well in advance of a detectable HD-like behavioral phenotype. However, reduced excitatory synaptic activity is observed in the stratum in later disease stages when a behavioral phenotype is present (Joshi et al., 2009). Synapse instability leading to synapse loss is well documented in HD, particularly in later disease stages (Murmu et al., 2013, 2015; Smith-Dijak et al., 2019). Interestingly, a similar bidirectional alteration in excitatory synaptic activity was observed in a conditional wtHTT knockout model (McKinstry et al., 2014). By crossing Emx1-Cre mice with wtHTT floxed mice, the authors generated mice with wtHTT depleted in the developing cortex. In these mice, excitatory synaptic connections developed in an accelerated manner in both the cortex and the striatum, although the accelerated synaptogenesis could not be maintained in the cortex and by 5 weeks of age, a reduction of synaptic contacts was observed. Thus, evidence for early hyperexcitability followed by synapse loss has been observed for both HD and wtHTT loss. A separate study observed axonal degeneration when wtHTT was deleted from forebrain neurons (Dragatsis et al., 2000). A similar approach was used more recently to deplete wtHTT in striatal SPNs during development (Burrus et al., 2020). By crossing A2A-Cre or D1-Cre mice with wtHTT floxed mice, the authors generated mice that lack wtHTT in either direct pathway SPNs (dSPNs) or indirect pathway SPNs (iSPNs). When they examined key projection targets of SPNs, namely the globus pallidum external segment (GPe) and the substantia nigra pars reticulata (SNR), they found that wtHTT was required for the proper development of inhibitory synaptic connections. Knocking out wtHTT in iSPNs decreased the number of inhibitory synapses and reduced mIPSC frequency in the GPe. Knocking out wtHTT in dSPNs increased the number of inhibitory synapses in the GPe (but was without effect on mIPSC frequency) and increased mIPSC frequency in the SNR (but was without an effect on inhibitory synapse number). In addition to altering inhibitory synapse development in key SPN target regions, wtHTT knockdown in either dSPNs or iSPNs was sufficient to impair motor function. Together, these studies clearly indicate that wtHTT plays a key role in proper synapse development. As HD mutation carriers are born with low levels of wtHTT in addition to the presence of mHTT, it is possible that the low wtHTT expression, or dominant-negative functions of mHTT presence, may alter the normal course of synapse development. Whether similar alterations in synaptic connectivity are observed following wtHTT reduction in adulthood remains to be seen, though particular attention must be paid in future studies to assess whether synapse loss can result from long-term HTT reduction therapies.

## Huntingtin Loss in Adulthood: Is It Safe?

One particularly pressing question that remains to be answered is whether or not wtHTT loss in adulthood is safe. Although many *in vivo* studies have been performed that reduce wtHTT to varying amounts in the brain, a clear consensus has yet to be reached (for a detailed recent review on HTT-lowering, also see Grondin and Kaemmerer, 2019). Methodological differences make it extremely challenging to directly compare the results of independent studies, as the magnitude, spread and duration of wtHTT loss can vary considerably from one study to the next. Furthermore, the readout of tolerability can range from survival to rotarod performance to western blot and immunohistochemical quantification of markers of neuronal density and gliosis. Based on our review of the literature, it is clear to us that wtHTT loss is not well-tolerated at the synaptic level, which is known to precede neurodegeneration in not just HD but also in many other neurodegenerative diseases (Selkoe, 2002; Milnerwood and Raymond, 2010; Mandolesi et al., 2015; Tyebji and Hannan, 2017). While the present review focused on wtHTT’s role at the pre- and postsynapse, it is well-established that many synapses exist in a tripartite configuration with astrocytes playing an essential role in synaptic function. For example, perisynaptic astrocytic processes dictate the spatial spread and temporal profile of extracellular glutamate transients following synaptic release (for a recent review, see Brymer et al., 2021). Astrocyte dysfunction has been well-documented in HD (Khakh et al., 2017), and expressing mHTT specifically in astrocytes can impair astrocytic BDNF release (Hong et al., 2016) and recapitulate many key features of the HD-like phenotype in mice (Bradford et al., 2009). Conversely, reducing mHTT in astrocytes can ameliorate behavioral, electrophysiological and neuropathological measures of disease progression in HD mice (Wood et al., 2019). At present, very little is known regarding how wtHTT loss affects essential astrocytic functions; however, given that mHTT inhibits astrocytic BDNF release by reducing BDNF vesicle docking, and given the known roles of wtHTT in vesicular regulation described earlier in this review, it would not be surprising if wtHTT loss in astrocytes exerted a similar effect. Given the strong link between astrocyte dysfunction and HD pathogenesis, and the non-selective nature of many HTT-lowering therapeutics, it is important that we better understand the role of wtHTT in not just neurons, but glial cells as well.

Numerous studies in both rodents and Rhesus moneys have reported that partial reduction of wtHTT (ranging from approximately 40 to 75% loss) does not negatively impact motor function nor does it cause any obvious signs of neurodegeneration, gliosis or inflammation (McBride et al., 2011; Grondin et al., 2012; Kordasiewicz et al., 2012). In the HD-171-82Q mouse model of HD, reducing both wtHTT and mHTT significantly improved the motor phenotype in these mice (Boudreau et al., 2009). Similarly, a study using tamoxifen-induced Cre recombinase demonstrated that wtHTT depletion in the adult brain had no effect on motor performance on the rotarod 7-8 months after tamoxifen injection (Wang et al., 2016). In this same study, 3 months of wtHTT knockdown had no effect on brain size nor did it affect the expression of markers of neurons, astrocytes, apoptosis or autophagy. On the other hand, RNAi-mediated wtHTT reductions in the striatum have been shown to alter the expression of numerous genes; genes that are up- or downregulated by wtHTT loss in adulthood play key roles in many of the functions described above in the present review, including axonal transport and secretion, LTP and LTD, glutamate and calcium signaling, and synaptic neurotransmission (Boudreau et al., 2009; Drouet et al., 2009).

Huntington disease mutation carriers almost always carry one copy of each the mutant allele and the wild-type allele. As a result, wtHTT levels are reduced throughout development and will be reduced further with any of the non-selective HTT reduction strategies. wtHTT reduction during development is likely to exert a negative effect on its own, as wtHTT heterozygous mice exhibit motor and cognitive deficits as well as neurodegeneration (Nasir et al., 1995; O’Kusky et al., 1999). Interestingly, in HD mutation carriers, a SNP on the wt*HTT* allele that reduces its expression is associated with an earlier disease onset (Bećanović et al., 2015). Recently, Dietrich and colleagues (2017) used a tamoxifen-inducible Cre-recombinase model to deplete wtHTT. However, in their study, one allele of wt*HTT* was floxed while the other was knocked out. In other words, these mice developed with half the normal levels of wtHTT before wtHTT was depleted by tamoxifen injection in adulthood at 3, 6, and 9 months of age. When wtHTT was depleted in adulthood in these mice, the authors observed a significantly reduced lifespan, impaired motor performance on the rotarod, various behavioral abnormalities (as assessed by the SHIRPA scale described previously Glynn et al., 2003), reduced brain weight, reactive gliosis, and impaired iron metabolism leading to calcification in the thalamus (Dietrich et al., 2017). Despite all these observed consequences of wtHTT loss, they found no clear evidence of pathology in the striatum or cortex, again suggesting that the regional sensitivities of wtHTT LOF do not necessarily mimic those of mHTT gain of function.

## Conclusion

Many of the studies discussed in this review demonstrate a clear role for wtHTT in synaptic function; for example, by complexing with essential synaptic proteins or by facilitating the delivery of synaptic cargoes from the cell body to pre- and postsynaptic compartments. In studies that reduce wtHTT *in vivo*, many putative emerging synaptic deficits are likely to go unnoticed in behavioral assessments that occur within a few months of wtHTT knockdown. After all, an excessive number of CAG repeats — only seen in the most severe cases of juvenile HD — is required to produce a HD-like phenotype within the lifespan of a rodent. In HD, synaptic deficits emerge before cells die and before a behavioral phenotype is evident (Li et al., 2003; Milnerwood and Raymond, 2010; Raymond et al., 2011; Tyebji and Hannan, 2017; Ravalia et al., 2021). Here, we argue that many core synaptic functions are at risk with non-selective HTT reduction strategies, and that if those synaptic functions are indeed compromised following wtHTT knockdown, then it is only a question of how long it will take before overt consequences are observed at the behavioral level. While we are not arguing against the belief that the net benefit of combined wtHTT and mHTT lowering may outweigh the continued presence of high levels of mHTT in HD individuals, we do argue that longer durations of wtHTT loss coupled with more sensitive tests of CNS dysfunction are required before a consensus can be reached regarding the relative tolerability of wtHTT loss in adulthood. Lastly, we emphasize the importance of acknowledging that the most sensitive regions in HD, such as the striatum and cortex, are not necessarily the regions that will be impacted the most by wtHTT reduction. For example, the thalamus is rarely discussed as a major brain region of particular susceptibility in HD yet shows striking calcification following wtHTT loss (Dietrich et al., 2017). Iron homeostasis is altered in the thalamus and the cerebellum following wtHTT loss (Dietrich et al., 2017), even though the cerebellum is often relatively spared in HD mouse models (Zhang et al., 2010; Carroll et al., 2011; Brooks et al., 2012). Similarly, the regional cell death patterns observed in heterozygous wtHTT knockout mice do not match the patterns of cell death observed in HD (Nasir et al., 1995; O’Kusky et al., 1999). Thus, when assessing the consequences of wtHTT loss, it is of utmost importance to consider extrastriatal brain regions as well as non-motor behavioral signs, as the non-motor symptoms of HD can oftentimes become the most burdensome aspect of the disease. Interestingly, there is evidence to suggest that wtHTT function can modulate depression and anxiety, two debilitating non-motor symptoms associated with HD. Specifically, reduced phosphorylation of wtHTT at S1181 and S1201 can increase hippocampal neurogenesis and reduce anxiety and depression-like behaviors in mice (Ben et al., 2013). By obtaining a more thorough understanding of the consequences of wtHTT loss, it will be possible to determine whether wtHTT can be safely lowered in HD patients, and if not, what essential functions of wtHTT must be restored following non-selective HTT reduction.

## Author Contributions

JB, EH, and MP wrote the manuscript. All authors contributed to the article and approved the submitted version.

## Conflict of Interest

The authors declare that the research was conducted in the absence of any commercial or financial relationships that could be construed as a potential conflict of interest.
